# The Efficacy of Backward Walking on Static Stability, Proprioception, Pain, and Physical Function of Patients with Knee Osteoarthritis: A Randomized Controlled Trial

**DOI:** 10.1155/2021/5574966

**Published:** 2021-06-11

**Authors:** Zehua Chen, Xiangling Ye, Yi Wang, Zhen Shen, Jiatao Wu, Weijian Chen, Tao Jiang, Huai Wu, Xuemeng Xu

**Affiliations:** ^1^The Fifth Clinical Medical College of Guangzhou University of Chinese Medicine, Guangzhou 510405, China; ^2^Kunming Municipal Hospital of Traditional Chinese Medicine, Kunming 650011, China; ^3^Guangdong Second Traditional Chinese Medicine Hospital, No. 60 Hengfu Road, Guangzhou, Guangdong 510405, China

## Abstract

**Objective:**

Impaired static stability and proprioception have been observed in individuals with knee osteoarthritis (KOA), which serves as a major factor increasing risk of fall. This study aimed to investigate the effects of backward walking (BW) on static stability, proprioception, pain, and physical function in KOA patients.

**Methods:**

Thirty-two subjects with knee osteoarthritis were randomly assigned to either an BW group (BG, *n* = 16) or a control group (CG, *n* = 16). The participants in the BG received combination treatment of a 4-week BW training and conventional treatments, while those in the CG was treated with conventional treatments alone. All the participants were tested for the assessment of static stability [center of pressure (COP) sway, including sway length (SL, mm) and sway area (SA, mm^2^)] and proprioception [average trajectory error (ATE, %) and completion time (CT, second)]. Additionally, pain and knee function scores were measured by the numerical rating scale (NRS) and the Western Ontario and McMaster Universities Osteoarthritis (WOMAC) Index, respectively. The assessments were conducted before and after intervention.

**Results:**

The COP sway (SA and SL), ATE, NRS, and WOMAC showed a significant decline at week 4 in the two groups in contrast to their baseline (*P* < 0.05). Moreover, after 4-week intervention, the SA [(610.50 ± 464.26) mm^2^ vs. (538.69 ± 420.52) mm^2^], NRS [(1.56 ± 0.63) vs. (2.25 ± 0.86)], and WOMAC [(11.69 ± 2.50) vs. (16.19 ± 3.94)] showed a significantly greater decrease in the BG compared to the CG (*P* < 0.05, respectively). However, the proprioception (ATE and CT) was closely similar between both groups at week 4 (*P* > 0.05).

**Conclusion:**

BW is an effective adjunct to conventional treatment in reducing pain, improving physical function and static stability for KOA patients. It should be taken into consideration when developing rehabilitation programs for people with KOA.

## 1. Introduction

Knee osteoarthritis (KOA), as a common disease, heavily compromises the health of the elderly. With the growing population of obesity and aging, the prevalence of KOA will become higher, which has been a serious global health concern [1]. Individuals with KOA always demonstrate severe symptoms including poor balance [2], stability deficits [3, 4], and impaired proprioception [5], in addition to joint swelling, pain, stiffness, muscle weakness, deformity, reduced joint motion, and disability [1]. Posture control is viewed as a key factor for the incidence of falls. Imbalance in the center of gravity of the body could reduce stability and increases the risk of falls [6], which would result in bone fractures or fatal injuries for older adults. Meanwhile, proprioception could influence the ability of limb coordination, which played a great role in postural control [7]. Therefore, proprioception impairment was harmful to the balance of skeletal muscles around the knee joint and increased the risk of falling [3]. As was reported, pain served as an important factor for postural sway, proprioception, and quadriceps strength in subjects with KOA [8, 9]. Medical treatments could alleviate symptomatic pain and consequently contribute to be benefit for the improvements of balance, posture stability, and proprioception [10, 11]. However, medications had some limitations in the clinical practice due to the side effects [12]. Thus, it is critical and urgent to explore a safe, effective, and feasible therapy to improve balance, stability deficits, and impaired proprioception.

In recent years, with the continuous exploration of clinical practices for the treatment of KOA, many complementary and alternative medicine methods have been developed, such as Tai chi [13], herbal remedies [14–16], and Baduanjin [17]. Backward walking (BW) training is recently introduced as a physiotherapy treatment for KOA patients, and several studies [18–20] suggested that a BW program exerts an impact on pain, functional disability, quadriceps muscle strength, and performance in the patient with KOA. The latest meta-analysis [21] showed that BW, as an adjunctive therapy, with conventional treatment was effective and worth to promote in patients with KOA. Furthermore, current evidence reveals that it had been considered as a potential strategy to improve balance performance and prevent falling for health subjects [22] and the people suffering from stroke [23], or cerebral palsy [24]. It was previously proved that significant improvements on balance and gait were observed after 4-week BW training [22, 25, 26].

Until now, only one study has reported that, for KOA patients, BW has benefit for balance improvement evaluated by using a subjective scale. The effects of BW on static stability and proprioception for patients with knee osteoarthritis are still unreported. Consequently, the aim of the present study is to investigate whether the pain, physical function, postural stability, and proprioception of KOA patients could be improved following a 4-week BW intervention using a randomized controlled trial (RCT).

## 2. Methods

### 2.1. Study Design

This research was designed as a pilot RCT to explore the effect of BW on postural stability and proprioception in patients with KOA. It was carried out at the Guangdong Second Traditional Chinese Medicine Hospital from September 15, 2019, to May 15, 2020. Ethical approval was obtained from the Ethics Committee of Guangdong Second Traditional Chinese Medicine Hospital (no. E1949) and it was registered at the China Clinical Registration Center (Registration no. ChiCTR1900026400). In this study, all included participants provided written informed consent and could withdraw from the study at any time.

### 2.2. Participants

A total of 48 participants with KOA diagnosed by the American College of Rheumatology clinical criteria [27] were enrolled from outpatients of the hospital. The other inclusion criteria were (i) age from 50 to 75 years, (ii) Kellgren/Lawrence [28] (K/L) grade ≥1 in one or both knees, (iii) no balance training experience, such as Tai Chi, Baduanjin, and Yoga, prior to six months before enrollment, and (iv) an ability to stand independently on the platform for 30 seconds without any assistive device for static stability test and depict 5 circles within 120 seconds for the proprioception assessment. The exclusion criteria were (i) presence of any known inflammatory rheumatic disease/arthritis; (ii) concomitant neurologic diseases, such as stroke, Parkinson's disease, severe cardiovascular, respiratory, spinal cord injury, or other musculoskeletal diseases; (iii) presence of acute joint effusion in knees [29]; (iv) use of any medications that could affect the musculoskeletal system or postural stability; and (v) history of ankle diseases and lower extremity fracture/surgery.

The included patients in the study were randomly assigned to either a BW group (BG) or a control group (CG) in a 1:1 ratio by using a balanced randomization method in accordance with the random number table. The numbers were kept at a locked location in a sealed, opaque envelope, to be later opened on the participants' agreement to participate.

### 2.3. Interventions

The included participants received conventional treatment comprising acupotomy, medications, and routine exercise, once a week for 4 weeks. Based on the previous method [30], the subjects in both groups were treated with needle-knife (Hanzhang Acupotome; Beijing Huaxia Acupotome Medical Equipment Factory, Beijing, China) therapy at the dominant inserted points of Neixiyan (Ex-LE4) and Waixiyan (Ex-LE5), as well as the conjugate points Dubi (ST35) and Xuehai (SP10). The prescribed acupotomy treatment was performed by an experienced therapist (XM Xu, a Chief Physician with 30-year clinical experience) for the participates, once a week for 4 weeks. All of the patients were prescribed with an oral medication, Celebrex capsules (Pfizer, H20140106, 0.2 g/d, once a day), for the first 6 days, while no extra painkillers were used in the next 3 weeks. Additionally, straight leg raising, as a routine exercise, was prescribed to practice at home for both legs, 1 set of 10 repetitions, twice a day, and gradually increase exercise time to 3 sets over the 4-week period, according to their pain intensity (pain score＜3) evaluated by using numerical rating scale (NRS) [31].

Participants allocated to the CG received the acupotomy therapy and completed the routine exercise as mentioned above. Participates in the CG were asked to maintain their daily habits and were discouraged from taking any other exercise. Patients allocated to BG were required to take part in BW training, in addition to the conventional treatment as the same treatment as the patients in the CG. According to the previous training program [18], BW program consisted of 10 min of BW training with 5-min warm-up and cool-down sessions 3 days a week for 4 weeks at their comfortable walking speed. Participates took the BW training session in the hospital for the first time under the supervision of another therapist (ZH Chen). After the initial training in hospital, the participants were instructed to continue to practice at home for the remaining time (till week 4) and gradually increase their walking time up to 30 min over the 4-week period, if they did not obtain an increasing pain score (NRS＜3). All participants were reminded and checked up via telephone.

### 2.4. Outcomes

The demographic characteristics were collected at baseline. Static stability, proprioception, NRS, and the Western Ontario and McMaster Universities Osteoarthritis (WOAMC) Index [32] were determined by a trained therapist (WJ Chen) who was blinded to the group allocation during evaluations at two time points: baseline (week 0) and week 4.

#### 2.4.1. Assessment of Static Stability and Proprioception

The parameters of center of pressure (COP) were always measured to assess postural stability, which served as an assessment for postural stability [4]. During the measurement, the participate was required to stand statically with both legs on the Dynamic and Static Balancing Instrument (Pro-kin 254P, TecnoBody Company, Italy) for 30 seconds, and COP sways including COP sway length (SL, mm) and sway area (SA, mm^2^) were documented automatically. The participants were tested with open eyes and their upper limbs placed on the side of body. As was reported, the smaller value of COP sways (SL and SA) revealed the better postural stability [5].

Proprioception measurement was conducted on the same machine. The participates were required to depict 5 circles (the left foot in a counterclockwise direction and the right foot in a clockwise direction) along the trajectory specified in the prescribed time (120 seconds), as was prompted by the system. Additionally, the subjects were administrated to complete the task with the fastest speed and the best accuracy. During proprioception testing, the participants' upper limbs were placed on the handrail of the machine. The average trajectory error (ATE, %) and completion time (CT, second) was recorded for the measurement of proprioception [33]. The smaller ATE meant more accurate proprioception; and shorter CT represented better proprioception.

Prior to testing, participants were asked to familiarize themselves with the testing process and conduct two simulation tests. Sufficient rest periods were given between trials. All participants were tested by the same researcher (WJ Chen) in the same way, requiring the test environment to be quiet and the body to maintain a standard position.

#### 2.4.2. Assessment of NRS and WOMAC Score

Pain and knee function score measured for the participates by using the NRS and the WOMAC, respectively, were assessed at baseline and week 4. NRS, a self-rated scale, indicates the level of pain (0 = no symptoms; 10 = extreme symptoms). WOMAC index comprises 3 components (24 items in total), pain (5 items), stiffness (2 items), and function (17 items). Each item graded in a numerical rating scale ranges from 0 (“none”) to 4 (“extreme”), and the total score of the 24 items is 96 (pain: 20; stiffness: 8; function: 68).

#### 2.4.3. Safety Record

Any occurrences of adverse events during the study would be recorded, and the affected participate would be instructed to discontinue the treatment. Meanwhile, necessary measures would be taken to deal with the adverse events.

### 2.5. Statistical Analysis

The required sample size was determined taking as a reference the data (effect size = 0.59, 1-*β* = 0.80, *α* = 0.10) described by Burcal et al. [34]. We performed the statistical analyses by using SPSS 25.0 (IBM Corp., NY, USA) software. Shapiro-Wilk test was used to assess normality for continuous characteristics. Based on the result of normality assessment, *T* test or nonparametric test (Mann-Whitney) was preformed to assess the differences between two groups. The categorical variables were assessed by chi-square test for between-group comparison. Comparing the proprioception and COP sway parameters before versus after intervention between intragroup, the paired Student's t-test was used for normal distribution; otherwise Wilcoxon Signed-rank test was used. Two-way repeated measures analysis of variance (RM ANOVA) (group × time) was employed to examine the interaction effect between group and time. If a significant interaction was detected, Student's t test for unpaired or paired data was employed. All continuous variables were presented as mean ± standard deviations. Statistical significance was accepted at *P* < 0.05.

## 3. Results

### 3.1. Participants Characteristics

**Figure 1:**
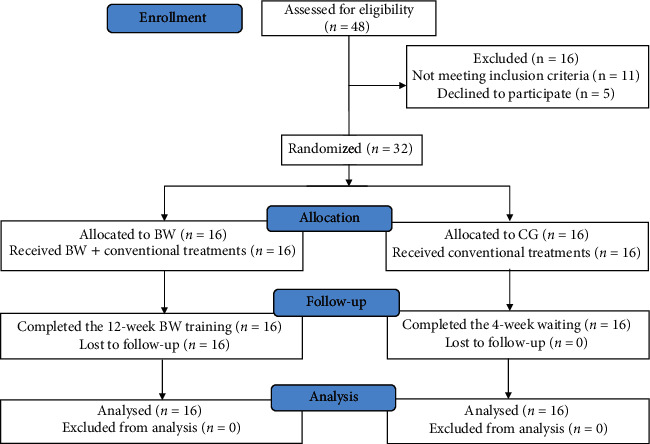
The flow chart of the participants in the study.

A total of 32 patients were included in this study after being screened against the selection criteria. Finally, thirty-two included participates were randomly assigned to the BG (3 males and 13 females) or the CG (3 males and 13 females). The flow chart of the participants of this RCT was illustrated in Figure 1. The age, gender, weight, height, body mass index (BMI), and K/L of the two groups were closely similar. Baseline demographics of both groups are presented in Table 1.

### 3.2. Static Stability

**Figure 2:**
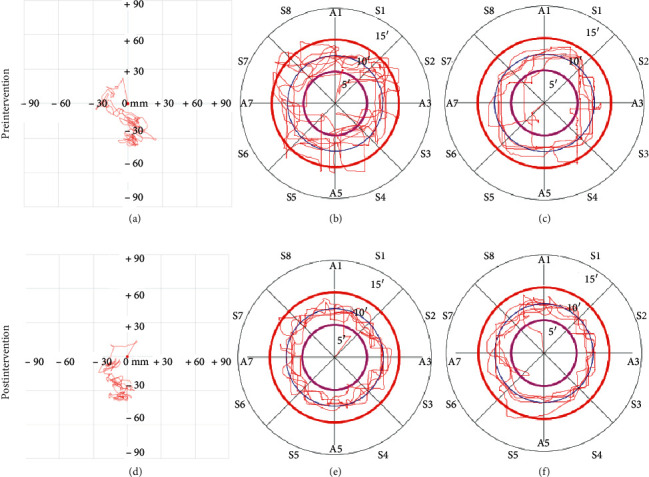
Example of proprioception and COP sway path tests for before and after intervention. (a) COP sway before intervention, (b) proprioception in left leg before intervention, (c) proprioception in right leg before intervention, (d) COP sway after intervention, (e) proprioception in left leg after intervention, and (f) proprioception in right leg after intervention.

Table 2 displays pre- and postintervention values regarding SL and SA. At baseline, no significant difference was found between the two groups. The results of the present study showed that no significant group × time interaction effects in SL (*F* = 2.063, *P*=0.156, *η* [2] = 0.033) and SA (*F* = 1.075, *P*=0.304, *η* [2] = 0.018) were found. Significant decline was observed in SA and SL between the pre- and posttreatment measurements of the BG, *P* < 0.01 and *P* < 0.01, respectively. Moreover, BG had a significantly greater reduction than CG in SA (mean changes, 339.6 versus 90.31; *P*=0.013). The example of COP sway before and after intervention was illustrated in Figures 2(a) and 2(d).

### 3.3. Proprioception

At baseline, ATE and CT were closely similar between BG and CG. As was shown in Table 3, no group × time interaction effect was found in ATE and CT in both legs of the two groups. After 4-week intervention, BG and CG showed a significant reduction in ATE on left (*P*=0.045 and *P*=0.003, respectively) and right leg (*P*=0.003 and *P*=0.002, respectively) between before and after intervention, whereas the improvement in CT on both legs was not examined. However, there was no significant difference in ATE and CT at week 4 on left (*P*=0.312 and *P*=0.136, respectively) and right (*P*=0.171 and *P*=0.451, respectively) legs between both groups. Furthermore, the improvements in ATE on both legs remained closely similar between the two groups. The example of the comparison of ATE and CT in both legs before and after intervention was shown in Figures 2(b), 2(c), 2(e), and 2(f).

### 3.4. NRS and WOMAC

Table 4 detailed the outcome assessment at 4 weeks at the end of trial completion. There was no significant difference in NRS and WOMAC between intergroups before intervention, whereas a significantly lower NRS and WOMAC score were observed in the BG than those in the CG after intervention (*P*=0.02 and *P*=0.001, respectively). Significant group × time interaction effects were found in WOMAC (*F* = 4.667, *P*=0.035, *η* [2] = 0.072) and function (*F* = 5.363, *P*=0.024, *η* [2] = 0.082). Results from the simple effect test indicated that, compared to the baseline, a significant decrease in NRS and WOMAC was determined in the two groups at week 4. Regarding pain and function, compared to the baseline, BG showed a significant improvement in them after 4-week intervention, whereas pain relief was not obviously examined in the CG. Most importantly, a significantly greater reduction in NRS, WOMAC, pain, and function was observed in the BG in comparison to the CG (*P*=0.048, *P*=0.013, *P*=0.019 and *P*=0.002, respectively).

### 3.5. Safety Report

In this trial, no adverse event was reported in the two groups during the 4-week intervention period. In the CG, one patient still suffered from a moderate activity pain (NRS = 4) at week 4, and then he received the intra-articular injection of sodium hyaluronate and the pain gradually subsided.

## 4. Discussion

Static stability is considered to be one key predictor of falls among the elderly population. COP parameters measured by using force plate were always applied to assess the static postural stability, which was proved to present excellent reliability [35]. Lots of factors attribute to stability impairment, such as age, muscle strength, proprioception, axial alignment of the lower extremity, and even knee sleeve [36]. It was reported that people suffering from KOA showed static stability deficit [37]. Moreover, our previous study [5] suggested that foot posture was closely associated with static postural control. Recently, increasing number of studies reported the benefits of BW for balance improvement. The present randomized, controlled trial investigated the effect of BW on static stability, proprioception, pain, and function in patients with knee osteoarthritis. The results of this study showed that SA, NRS, WOMAC, pain, and function had a significantly greater change after 4-week intervention in the BG than those in the CG, which revealed that, compared to the CG treated with conventional methods alone, BW as an adjunctive intervention in coordination with conventional treatments had a more favorable effect on static stability enhancement, pain relief, and function improvement in KOA patients. However, even though BG and CG showed a significant improvement in proprioception, the advantage of BW was not obviously observed for proprioception improvement by comparing the two groups.

BW, unsimilar to forward walking, requires specialized control circuits, in addition to rhythm circuitry [38]. The toes contact the ground first and the heel is lifted off the ground at the end during BW stance phase, which leads to different muscles activation patterns and gait characteristics. Motor systems could initiate timely, then appropriate, responses and consequently counteract various disturbances [39], contributing to achievement of equilibrium condition through modifying the biomechanical state. BW training caused changes in movement control system and gait characteristics and exerted a positive effect on postural stability. Furthermore, because of little dependence on vision, BW training participants had to rely more on neuromuscular proprioceptive and vestibular senses to maintain postural stability [40]. It was proved that BW training is more effective in improving gait speed and stride length [41]. In addition, it was previously reported [18–20, 26, 42] that BW could reduce pain, increase quadriceps muscle strength, enhance hamstring flexibility, and improve physical function for individuals with KOA. Gondhalekar et al. [19] indicated that after a minimal effective dosage of 3 weeks, combination of BW and the routine physiotherapy significantly improved function in KOA patients. Those findings were in agreement with the results regarding NRS, WOMAC, pain, and function in the present study.

As a simple, practicable, and effective training, BW was used to improve the balance performance for stroke [25] and children with hemiparetic cerebral palsy [34], and the favorable effects of BW on proprioception in nonathletic males [43] and subjects with anterior cruciate ligament reconstruction [44] were observed. Nevertheless, to the best of our knowledge, this is the first time to evaluate the effect of BW on static stability and proprioception in people affected by KOA. With regard to SA, we found that both groups showed a significant reduction, and there was a significantly greater change in the BG after a 4-week BW intervention period than the waitlist control, which echoed the recently published meta-analysis [40]. However, after 4-week intervention, BG had no significant decrease in SL compared to before intervention, and there was on significant difference in SL between the two groups. It seemed that obvious difference was more likely to be detected in SA rather than SL. Similarly, Ye J et al. [17] thought SA was more sensitive in terms of reflecting a postural stability than SL. Additionally, better proprioception was examined both in BG and CG, but the improvement of proprioception was similar between BG and CG, which meant the effect of BW on proprioception was unobvious. This result was not the same as the results reported by Sedhom et al. [43] and Shen M et al. [44]. On one hand, proprioception improvement in the two groups should be attributed to acupotomy and routine exercise. It had been proved that acupotomy was beneficial to reduce pain and improve joint function for KOA patients [45], which resulted in a better proprioception before intervention. On the other hand, the proprioception mainly includes the sense of position and movement and the sense of effort, force, and heaviness [46], and tendons and muscle spindles are the two major mechanoreceptors [47]. As was reported, muscle weakness or atrophy appeared in patients with OA as one of the earliest symptoms [48]. Due to the muscle problem, it could be difficult to obtain proprioception recovery for the KOA patients, which might be the reason for the results that patients in the BG showed no significant improvement in proprioception more than those in the CG at postintervention week 4, or a longer term of BW intervention was required. Of note, the results derived from this study showed that, at postintervention week 4, the SA presented no significant difference between BG and CG, whereas a significantly bigger change of it was observed in the BG than those in the CG when it was compared between before and after the intervention. It could be explained by the high intragroup variability in SA, which resulted in no significant intergroup difference before and after intervention. Nevertheless, changes of SA, NRS, WOMAC, pain, and function in the BG were significantly larger than those in the CG, which was obvious enough to prove the superiority of BW combined with conventional treatments for enhancing static stability, reducing pain, and improving function in KOA patients compared to conventional treatments used alone.

There are some limitations in the present study: firstly, even though 4-weeks BW intervention was proved to be the effective dosage for pain, function, and balance in KOA patients, a longer intervention period and follow-up might bring greater changes in the outcomes; secondly, the small number of cases was included because it was difficult to complete the task of proprioception test for the KOA patients with impaired proprioception, so a more practicable and easier method for proprioception measurement would be helpful to conduct a clinical trial with larger scale; thirdly, the included patients showed a high variability in SA which resulted in unobvious benefits from BW for KOA patients after intervention; hence, inclusion criteria should have restrictions on static stability in future study.

## 5. Conclusion

In conclusion, the current study indicated that, compared to the waitlist control, KOA patients treated with 4-week BW training in combination with conventional treatment showed a greater reduction in pain and functional disability and had a greater improvement in static stability. However, for KOA patients, 4-week BW combined with conventional therapy presented no significantly greater improvement in proprioception than conventional therapy used alone.

## Figures and Tables

**Table 1:** Characteristics of the participants in the study.

	BG (*n* = 16)	CG (*n* = 16)	*P*
Age (years)	60.31 ± 7.85	60.94 ± 6.89	0.812
Gender (male/female)	3/13	3/13	
Height (cm)	160.44 ± 6.49	160.75 ± 7.16	0.898
Weight (kg)	62.06 ± 7.62	63.06 ± 8.08	0.721
BMI (kg/m^2^)	24.08 ± 2.24	24.37 ± 2.29	0.721
K/L scale	3.38 ± 0.619	3.19 ± 0.66	0.410
Duration (month)	38.75 ± 38.32	35.75 ± 34.54	0.691

BG: backward walking group; CG: control group; BMI: body mass index; K/L : Kellgren/Lawrence.

**Table 2:** Comparison of static stability between the two groups over time.

Groups	Items	SL (mm)	SA (mm^2^)
BG	Before intervention	594.75 ± 205.13	949.56 ± 552.99
	After intervention	384.75 ± 106.99^△^	610.50 ± 464.26^△^
	Mean changes	−210.00	−339.06
CG	Before intervention	475.44 ± 156.72	629.00 ± 471.67
	After intervention	383.25 ± 171.88	538.69 ± 420.52^△^
	Mean changes	−67.19	−90.31^∗^
Time effect		0.079	0.01
Group◊Time effect	F	2.063	1.075
	P	0.156	0.304
	*η* ^2^	0.033	0.018

BG: backward walking group; CG: control group; SL: sway length; SA: sway area; ^△^intragroup difference before intervention, *P* < 0.05; ^∗^intergroup difference after intervention, *P* < 0.05.

**Table 3:** Comparison of proprioception between the two groups over time.

Groups	Items	Left side		Right side	
		ATE (%)	CT (s)	ATE (%)	CT (s)
BG	Before intervention	34.63 ± 13.20	85.94 ± 12.29	36.25 ± 11.58	85.88 ± 15.52
	After intervention	29.75 ± 8.07^△^	80.88 ± 8.28	28.19 ± 7.96^△^	85.88 ± 11.02
	Mean changes	−4.88 ± 13.62	−5.06 ± 9.72	−8.06 ± 9.04	0.00 ± 16.45
CG	Before intervention	34.06 ± 10.97	90.38 ± 17.88	34.19 ± 14.03	87.56 ± 19.52
	Afterintervention	27.06 ± 6.64^△^	88.19 ± 16.98	23.88 ± 9.39^△^	89.63 ± 12.93
	Mean changes	−7.00 ± 7.98	−2.19 ± 9.68	−10.31 ± 11.27	2.06 ± 15.43
Time effect		0.021	0.318	0.01	0.785
Group◊Time effect	F	0.179	0.160	0.168	0.075
	P	0.674	0.691	0.684	0.785
	*η* ^2^	0.003	0.003	0.003	0.001

BG: backward walking group; CG: control group; ATE: average trajectory error; CT: completion time; ^△^intragroup difference before intervention, *P* < 0.05; ^∗^intergroup difference after intervention, *P* < 0.05.

**Table 4:** Comparison of pain and function between the two groups over time.

Groups	Items	NRS	WOMAC			
			Total	Pain	Stiffness	Function
BG	Before intervention	3.69 ± 0.79	21.56 ± 6.18	5.63 ± 1.93	1.31 ± 1.58	14.63 ± 3.56
	After intervention	1.56 ± 0.63^△^	11.69 ± 2.50^△^	2.63 ± 0.81^△^	0.88 ± 1.09^△^	8.19 ± 1.87^△^
	Changes	−2.13 ± 1.09	−9.88 ± 4.99	−3.00 ± 1.67	−0.44 ± 0.73	−6.44 ± 3.69
CG	Before intervention	3.63 ± 0.96	21.13 ± 4.87	5.19 ± 1.56	0.94 ± 1.18	15.00 ± 3.31
	After intervention	2.25 ± 0.86^△^^*∗*^	16.19 ± 3.94^△^^*∗*^	3.31 ± 1.20	0.75 ± 0.93	12.13 ± 3.28^△^^*∗*^
	Changes	−1.38 ± 0.89^*∗*^	−4.94 ± 2.41^*∗*^	−1.88 ± 1.03^*∗*^	−0.19 ± 0.40	−2.88 ± 1.78^*∗*^
Time effect		0.001	0.001	0.001	0.309	0.001
Group◊Time effect	*F*	3.364	4.667	2.462	0.168	5.363
	*P*	0.072	0.035	0.122	0.683	0.024
	*η* ^2^	0.053	0.072	0.039	0.003	0.082

BG: backward walking group; NRS: numerical rating scale; WOMAC: the Western Ontario and McMaster Universities Osteoarthritis Index; ^△^intragroup difference before intervention, *P* < 0.05; ^*∗*^intergroup difference after intervention, *P* < 0.05.

## Data Availability

The data used to support the findings of this study are available from the corresponding author upon request.

## References

[1] Hunter D. J., Bierma-Zeinstra S. (2019). *Osteoarthritis. Lancet*.

[2] Hinman R. S., Bennell K. L., Metcalf B. R., Crossley K. M. (2002). Balance impairments in individuals with symptomatic knee osteoarthritis: a comparison with matched controls using clinical tests. *Rheumatology*.

[3] Hassan B. S., Mockett S., Doherty M. (2001). Static postural sway, proprioception, and maximal voluntary quadriceps contraction in patients with knee osteoarthritis and normal control subjects. *Annals of the Rheumatic Diseases*.

[4] Taglietti M., Dela Bela L. F., Dias J. M. (2017). Postural sway, balance confidence, and fear of falling in women with knee osteoarthritis in comparison to matched controls. *Physical Medicine and Rehabilitation*.

[5] Chen Z., Shen Z., Ye X., Wu J., Wu H., Xu X. (2020). Association between foot posture asymmetry and static stability in patients with knee osteoarthritis: a case-control study. *BioMed Research International*.

[6] Tinetti M. E., Speechley M., Ginter S. F. (1988). Risk factors for falls among elderly persons living in the community. *New England Journal of Medicine*.

[7] Henry M. (2019). Age-related changes in leg proprioception: implications for postural control. *Journal of Neurophysiology*.

[8] Hassan B. S., Doherty S. A., Mockett S., Doherty M. (2002). Effect of pain reduction on postural sway, proprioception, and quadriceps strength in subjects with knee osteoarthritis. *Annals of the Rheumatic Diseases*.

[9] Hall M. C., Mockett S. P., Doherty M. (2006). Relative impact of radiographic osteoarthritis and pain on quadriceps strength, proprioception, static postural sway and lower limb function. *Annals of the Rheumatic Diseases*.

[10] Cho H.-y., Kim E.-H., Kim J., Yoon Y. W. (2015). Kinesio taping improves pain, range of motion, and proprioception in older patients with knee osteoarthritis. *American Journal of Physical Medicine & Rehabilitation*.

[11] Kim D., Park G., Kuo L. T., Park W. (2018). The effects of pain on quadriceps strength, joint proprioception and dynamic balance among women aged 65 to 75 years with knee osteoarthritis. *BMC Geriatrics*.

[12] Persson M. S. M., Stocks J., Varadi G. (2020). Predicting response to topical non-steroidal anti-inflammatory drugs in osteoarthritis: an individual patient data meta-analysis of randomized controlled trials. *Rheumatology*.

[13] Zhang Z., Huang L., Liu Y., Wang L. (2020). Effect of Tai chi training on plantar loads during walking in individuals with knee osteoarthritis. *BioMed Research International*.

[14] Kakatum N., Pinsornsak P., Kanokkangsadal P., Ooraikul B., Itharat A. (2021). Efficacy and safety of sahastara remedy extract capsule in primary knee osteoarthritis: a randomized double-blinded active-controlled trial. *Evidence-Based Complementary and Alternative Medicine*.

[15] Askari A., Ravansalar S. A., Naghizadeh M. M. (2019). The efficacy of topical sesame oil in patients with knee osteoarthritis: a randomized double-blinded active-controlled non-inferiority clinical trial. *Complementary Therapies in Medicine*.

[16] Anvari M., Dortaj H., Hashemibeni B., Pourentezari M. (2020). Application of some herbal medicine used for the treatment of osteoarthritis and chondrogenesis. *Traditional and Integrative Medicine*.

[17] Ye J., Zheng Q., Zou L. (2020). Mindful exercise (Baduanjin) as an adjuvant treatment for older adults (60 Years old and over) of knee osteoarthritis: a randomized controlled trial. *Evidence-Based Complementary and Alternative Medicine*.

[18] Alghadir A. H., Anwer S., Sarkar B., Paul A. K., Anwar D. (2019). Effect of 6-week retro or forward walking program on pain, functional disability, quadriceps muscle strength, and performance in individuals with knee osteoarthritis: a randomized controlled trial (retro-walking trial). *BMC Musculoskelet Disorders*.

[19] Gondhalekar G., Deo M. (2013). Retrowalking as an adjunct to conventional treatment versus conventional treatment alone on pain and disability in patients with acute exacerbation of chronic knee osteoarthritis: a randomized clinical trial. *North American Journal of Medical Sciences*.

[20] Balraj Am., Krishnan R., Kamaraj B. (2018). Impact of retro-walking on pain and disability parameters among chronic osteoarthritis knee patients. *Physical therapy*.

[21] Balasukumaran T., Olivier B., Ntsiea M. V. (2019). The effectiveness of backward walking as a treatment for people with gait impairments: a systematic review and meta-analysis. *Clinical Rehabilitation*.

[22] Cha H.-G., Kim T.-H., Kim M.-K. (2016). Therapeutic efficacy of walking backward and forward on a slope in normal adults. *Journal of Physical Therapy Science*.

[23] Chen Z. H., Ye X. L., Chen W. J. (2020). Effectiveness of backward walking for people affected by stroke: a systematic review and meta-analysis of randomized controlled trials. *Medicine (Baltimore)*.

[24] Elnahhas A. M., Elshennawy S., Aly M. G. (2019). Effects of backward gait training on balance, gross motor function, and gait in children with cerebral palsy: a systematic review. *Clinical Rehabilitation*.

[25] Kim C.-Y., Lee J.-S., Kim H.-D. (2017). Comparison of the effect of lateral and backward walking training on walking function in patients with poststroke hemiplegia. *American Journal of Physical Medicine & Rehabilitation*.

[26] Manisha N., Joginder Y., Priyanka R. (2015). Effect of retro walking on pain, balance and functional performance in osteoarthritis of knee. *Indian Journal of Physiotherapy and Occupational Therapy - An International Journal*.

[27] Altman R., Asch E., Bloch D. (1986). Development of criteria for the classification and reporting of osteoarthritis: classification of osteoarthritis of the knee. *Arthritis & Rheumatism*.

[28] Kellgren J. H., Lawrence J. S. (1957). Radiological assessment of osteo-arthrosis. *Annals of the Rheumatic Diseases*.

[29] Hong B. Y., Lim S. H., Im S. A., Lee J. I. (2013). Effects of acute joint effusion on balance in patients with knee osteoarthritis. *American Journal of Physical Medicine & Rehabilitation*.

[30] Lin M., Li X., Liang W. (2014). Needle-knife therapy improves the clinical symptoms of knee osteoarthritis by inhibiting the expression of inflammatory cytokines. *Experimental and Therapeutic Medicine*.

[31] Thong I. S. K., Jensen M. P., Miró J., Tan G. (2018). The validity of pain intensity measures: what do the NRS, VAS, VRS, and FPS-R measure?. *Scandinavian Journal of Pain*.

[32] Ackerman I. (2009). Western Ontario and McMaster Universities osteoarthritis index (WOMAC). *Australian Journal of Physiotherapy*.

[33] Koutakis P., Mukherjee M., Vallabhajosula S., Blanke D. J., Stergiou N. (2013). Path integration: effect of curved path complexity and sensory system on blindfolded walking. *Gait & Posture*.

[34] Burcal C. J., Trier A. Y., Wikstrom E. A. (2017). Balance training versus balance training with stars in patients with chronic ankle instability: a randomized controlled trial. *Journal of Sport Rehabilitation*.

[35] Takacs J., Carpenter M. G., Garland S. J., Hunt M. A. (2014). Test re-test reliability of centre of pressure measures during standing balance in individuals with knee osteoarthritis. *Gait & Posture*.

[36] Chuang S.-H., Huang M.-H., Chen T.-W., Weng M.-C., Liu C.-W., Chen C.-H. (2007). Effect of knee sleeve on static and dynamic balance in patients with knee osteoarthritis. *The Kaohsiung Journal of Medical Sciences*.

[37] Pirayeh N., Shaterzadeh-Yazdi M.-J., Negahban H., Mehravar M., Mostafaee N., Saki-Malehi A. (2018). Examining the diagnostic accuracy of static postural stability measures in differentiating among knee osteoarthritis patients with mild and moderate to severe radiographic signs. *Gait & Posture*.

[38] Hoogkamer W., Meyns P., Duysens J. (2014). Steps forward in understanding backward gait. *Exercise and Sport Sciences Reviews*.

[39] Lawrence E. L., Cesar G. M., Bromfield M. R., Peterson R., Valero-Cuevas F. J., Sigward S. M. (2015). Strength, multijoint coordination, and sensorimotor processing are independent contributors to overall balance ability. *BioMed Research International*.

[40] Wang J., Xu J., An R. (2019). Effectiveness of backward walking training on balance performance: a systematic review and meta-analysis. *Gait & Posture*.

[41] Kraan G. A., van Veen J., Snijders C. J., Storm J. (2001). Starting from standing; why step backwards?. *Journal of Biomechanics*.

[42] Whitley C. R., Dufek J. S. (2011). Effects of backward walking on hamstring flexibility and low back range of motion. *International Journal of Exercise Science*.

[43] Sedhom M. G. (2017). Backward walking training improves knee proprioception in non-athletic males. *International Journal of Physiotherapy*.

[44] Shen M., Che S., Ye D., Li Y., Lin F., Zhang Y. (2019). Effects of backward walking on knee proprioception after ACL reconstruction. *Physiotherapy Theory and Practice*.

[45] Zhu J., Zheng Z., Liu Y. (2020). The effects of small-needle-knife therapy on pain and mobility from knee osteoarthritis: a pilot randomized-controlled study. *Clinical Rehabilitation*.

[46] Proske U., Gandevia S. C. (2012). The proprioceptive senses: their roles in signaling body shape, body position and movement, and muscle force. *Physiological Reviews*.

[47] Kharaji G., Nikjooy A., Amiri A., Sanjari M. A. (2019). Proprioception in stress urinary incontinence: a narrative review. *Medical Journal of the Islamic Republic of Iran*.

[48] Shorter E., Sannicandro A. J., Poulet B., Goljanek-Whysall K. (2019). Skeletal muscle wasting and its relationship with osteoarthritis: a mini-review of mechanisms and current interventions. *Current Rheumatology Reports*.

